# Revised Two-Stage Model of Preeclampsia Based on Autophagic Dysfunction: A Comprehensive Review

**DOI:** 10.3390/biom16030441

**Published:** 2026-03-15

**Authors:** Atsushi Furuta, Tomoko Shima, Takashi Nishigori, Kiyotaka Yamada, Haruka Nunomura, Mihoko Yoshida, Shina Sakaguchi, Takuya Majima, Akemi Yamaki-Ushijima, Kanto Shozu, Sayaka Tsuda, Shibin Cheng, Surendra Sharma, Akitoshi Nakashima

**Affiliations:** 1 Department of Obstetrics and Gynecology, University of Toyama, Toyama 930-0194, Japan; s0950074@med.u-toyama.ac.jp (A.F.);; 2 Department of Pediatrics, Warren Alpert Medical School of Brown University, Providence, RI 02903, USA; 3 Departments of Obstetrics and Gynecology and Pediatrics, University of Texas Medical Branch, Galveston, TX 77555, USA

**Keywords:** autophagy, TFEB, preeclampsia

## Abstract

A revised two-stage model of preeclampsia is proposed, centering on an autophagy-dependent requirement for extravillous trophoblast entry into the proximal one-third of the myometrium. The One-Third Myometrium Enigma, introduced here, denotes the unresolved physiological rule that early placentation requires trophoblasts to traverse decidua and reach the proximal one-third of myometrium under hypoxia and nutrient scarcity. The hypothesis posits a timed rise in basal autophagy to sustain trophoblast energy homeostasis and invasion, accompanied by TFEB-driven lysosomal programs that enable villous cytotrophoblast syncytialization. Autophagic dysfunction could contribute to shallow invasion, chronic placental hypoxia, fetal growth restriction, and release of placental injury signals preceding maternal syndrome. Potential failure modes include reduced autophagic flux due to inhibition of autophagosome to lysosome fusion or mistimed persistence of hypoxia signaling, such as prolonged HIF-1α activity. Collectively, this evidence suggests that impaired autophagy is a testable contributor to preeclampsia pathogenesis. Predictions include early risk stratification with circulating autophagy markers and extracellular vesicle microRNAs, and therapeutic benefit from autophagy modulation that targets AMPK or mTOR or activates TFEB with safety constraints. This framework reframes preeclampsia as a disorder of placental quality control and specifies where and when autophagy may be required.

## 1. Introduction

Proper placental development is crucial to the health of both the mother and the fetus. The placenta functions as the critical interface between maternal and fetal systems, mediating nutrient and oxygen exchange, waste removal, and immune tolerance. Disruption of this finely tuned process can result in placental malformation, a condition closely associated with adverse pregnancy outcomes. Among these outcomes, hypertensive disorders of pregnancy (HDP), including preeclampsia, are of major concern. Early-onset HDP with high risk of severity is often accompanied by placental malformation, contributing to fetal growth restriction (FGR), preterm birth, and even perinatal mortality [1]. HDP also poses significant risks to maternal health, leading to long-term cardiovascular complications, chronic kidney diseases, hyperlipidemia, diabetes, and progressive cognitive decline that may culminate in dementia, ultimately contributing to reduced life expectancy and, in severe cases, maternal death [2]. Despite extensive research, the mechanisms linking placental dysfunction to preeclampsia remain incompletely understood.

One mechanism receiving increasing attention is autophagy dysfunction in the placenta, contributing to both placental malformation and HDP pathogenesis. Autophagy is a conserved cellular process for degrading and recycling damaged organelles and proteins, crucial for cellular homeostasis under stress conditions such as hypoxia and nutrient deprivation. These conditions characterize the early placental environment in which, even under such harsh conditions, extravillous trophoblast (EVT) cells invade the maternal uterus to establish the maternal–fetal circulation, which is a hallmark of normal placental development in early pregnancy. Inadequate trophoblast invasion or differentiation into syncytiotrophoblasts (STBs) can impair placentation, leaving the maternal–fetal circulation incomplete and leading to systemic maternal endothelial damage via anti-angiogenic factors, which in turn can cause complications such as FGR and preeclampsia [3].

In the dynamic, low-oxygen environment of early placental development, trophoblasts must adapt to survive and invade the maternal tissue. Emerging evidence indicates that autophagy is a pivotal adaptive mechanism supporting trophoblast invasion under these harsh conditions [4,5,6]. Conversely, when autophagy is disrupted, trophoblast invasion into the maternal uterus is compromised, and the resulting shallow invasion gives rise to impaired placental development, which subsequently leads to chronic placental hypoxia [7]. This pathological state can serve as a trigger that drives the cascade of systemic maternal endothelial dysfunction characteristic of preeclampsia. We propose that autophagy inhibition contributes to the development of preeclampsia. However, previous reports present conflicting evidence, with some studies suggesting autophagy activation and others suggesting autophagy inhibition. Based on the existing literature, we have categorized studies reporting autophagy activation [8,9,10,11,12,13,14] (Table 1) and autophagy inhibition [7,15,16,17,18,19,20,21] (Table 2) in preeclampsia into two separate tables. This review aims to elucidate the role of placental autophagy dysfunction in the etiology of preeclampsia, which is categorized in the HDP subtype as a severe type, with a particular focus on clarifying how autophagy integrates cellular and molecular evidence with clinical observations. By explaining this mechanism, we hope to inform novel therapeutic strategies for managing preeclampsia and improving outcomes for both mothers and their offspring. Additionally, these insights highlight broader implications of autophagy research in reproductive medicine and maternal–fetal health.

## 2. How to Classify Multiple Etiologies of Preeclampsia

**Figure 1:**
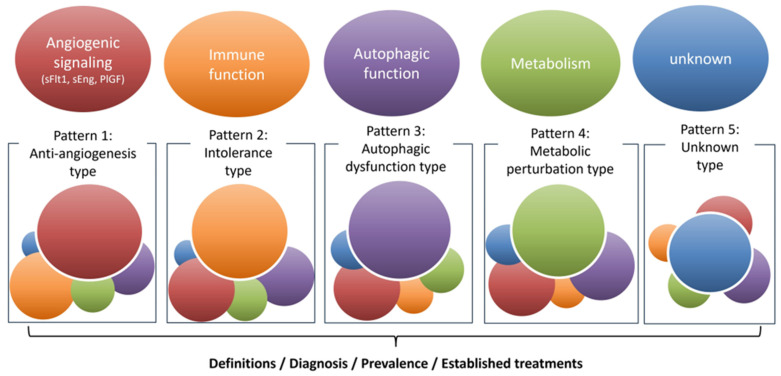
Etiologic subtypes of preeclampsia. This schematic classifies preeclampsia into etiologic patterns to enable subtype-specific diagnosis and therapy development. For each pattern, the framework highlights the need for a concise definition, practical diagnostics, and estimates of prevalence. Patterns depicted are: Pattern 1, anti-angiogenic type (therapeutic concept: lowering soluble Flt-1); Pattern 2, maternal intolerance type (therapeutic concept: immunomodulation or immunosuppression); Pattern 3, autophagic dysfunction type (therapeutic concept: pharmacologic upregulation of autophagy); Pattern 4, metabolic perturbation type (therapeutic concept: target the dominant metabolic epicenter); and Pattern 5, unknown type (discovery track). sFlt-1, soluble fms-like tyrosine kinase-1.

Clinically, early-onset preeclampsia and late-onset preeclampsia reflect syndromes in which different processes dominate. Typical early-onset cases, most often presenting between 20 and 29 weeks, arise from placental maldevelopment and display excess anti-angiogenic signaling. Key mediators include soluble fms-like tyrosine kinase-1 (sFlt1), frequently accompanied by soluble endoglin (sEng), together with other placenta-derived factors that disseminate systemically. Early-onset preeclampsia frequently coexists with FGR; progression to maternal injury in major organs such as the kidney, brain, and liver is common. In contrast, late-onset preeclampsia, defined by onset at 34 weeks’ gestation or later, is less often accompanied by overt placental hypoplasia or FGR; stronger correlations are seen with maternal metabolic risk, notably obesity and diabetes [22]. This two-group classification is helpful for clinical thinking, but it should not be viewed as a hard line. Many cases presenting between 30 and 34 weeks fall in between. It is therefore better to think of preeclampsia as a continuum in which placental and maternal factors contribute to different degrees. Furthermore, familial aggregation has been repeatedly demonstrated. Elevated risk is observed among first-degree relatives, and recurrence clusters within families across successive pregnancies. Genetic studies implicate multiple susceptibility loci distributed across maternal, fetal, and placental genomes [23,24,25,26]. The *FLT1* locus on chromosome 13q, first identified by a fetal genome-wide association study as genome-wide significant at rs4769613 with an additional independent signal at rs12050029, has been replicated, and functional work supports the same direction of effect, namely higher FLT1 expression in the placenta and increased circulating sFlt1 [27]. Aside from the *FLT1* locus, no single high-penetrance variant explains most cases; rather, modest-effect alleles accumulate and interact with immune and environmental factors. Therapeutic progress will require grouping these contributors, assigning weight to their relative impact, and deploying mechanism-based strategies, analogous to the evolution in oncology from organ-based cytotoxic regimens to pathway-targeted approaches. Within this framework, four pathogenic axes are particularly salient (Figure 1).

1. Anti-angiogenic signaling: placental overproduction of sFlt1, often accompanied by sEng, reproduces cardinal features of the syndrome in experimental systems and underpins clinical tools such as the ratio of sFlt1 to placental growth factor (PlGF), sFlt1/PlGF, for prediction and triage.
2. Immune dysregulation: epidemiological patterns point to incomplete maternal tolerance to paternal antigens. Primiparity, recurrence with partner change, and the heightened risk observed after oocyte donation or embryo transfer support this concept [28,29,30]. Mechanistic studies demonstrate impaired induction of regulatory T cells and lower expression of programmed cell death protein-1 on cytotoxic T cells, consistent with a pro-inflammatory decidual environment [31,32,33].
3. Metabolic perturbation: maternal diabetes and obesity confer risk. Reports describe lipid peroxidation, sphingolipid imbalance, reduced L-arginine to nitric-oxide bioavailability, and deficits in mitochondrial energy production; these observations identify metabolic stress as a disease amplifier and a source of candidate biomarkers and targets [34,35,36,37,38].
4. Autophagic dysfunction, proposed here as a fourth axis: autophagy complements the proteasome as a principal intracellular degradative system that supplies energy during scarcity and enforces protein and organelle quality control through selective pathways [39,40]. In addition, components of the autophagy machinery may contribute to unconventional protein secretion and exocytosis, as suggested by studies in non-trophoblast systems [41]; trophoblast-specific support remains limited. We propose that defective autophagy intersects with the axes above, compromises placental development, and escalates maternal endothelial stress.

In addition to the four axes outlined above, further determinants are likely to be identified. Because preeclampsia encompasses disease states in which one or more axes predominate, a subtype framework, such as that depicted in Figure 1, is warranted. This framework allows for the definition of discrete subtypes, the diagnosis of the dominant driver in each case, and the quantification of their relative prevalence, thereby enabling the development of subtype-specific treatment strategies. Mixed phenotypes are also expected, and some patients will exhibit overlapping contributions from several axes. These axes are further shaped by cross-cutting stress responses, including endoplasmic reticulum (ER) stress, oxidative stress, and hypoxia, as well as regulated cell death programs such as apoptosis, necrosis, pyroptosis, and ferroptosis [3]. Interactions among these processes converge to worsen placental function. Future work should establish standardized assessment tools for each axis and, in parallel, develop targeted interventions tailored to the dominant drivers in individual patients. Building on this framework, the next section examines the physiological limits on EVT invasion in early gestation and highlights autophagy as the adaptation that sustains invasive capacity.

## 3. The One-Third Enigma of the Myometrium in Normal Placental Development

Placental development begins with implantation of the blastocyst into the decidualized endometrium, followed by placental formation from the trophoblast, the outer cell layer of the blastocyst [42]. Trophoblast cells differentiate mainly along two pathways: into STBs, multinuclear cells that form the outer layer of placental villi to mediate nutrient exchange between mother and fetus, and into EVT, which invade maternal tissue to establish sufficient blood flow to the placenta [43]. A subpopulation of EVTs penetrates the uterine myometrium, the muscular layer of the uterus, where they remodel spiral arteries, thereby anchoring the placenta and ensuring an adequate maternal blood supply. Remarkably, EVT invasion in humans is highly controlled: these cells typically infiltrate about one-third of the myometrial depth and then stop. Considering that the uterine wall in the non-pregnant state is about 3 cm thick, invasion to one-third of its depth is a striking phenomenon that represents a unique feature of non-malignant trophoblasts. In contrast, cancer cells often invade without such limitation, leading to metastasis to other organs and ultimately causing death. We term this precise limitation the “One-Third Myometrium Enigma”, emphasizing our strong interest in the mechanism by which EVTs consistently halt after invading about 1 cm into the uterine wall. Notably, EVT behavior is reminiscent of malignant cell invasion in its depth and spread, but unlike cancer cells, trophoblast invasion is stringently regulated and self-limited. Crucially, EVT invasion occurs in an environment of extreme physiological stress. Until 8–10 weeks of gestation, the maternal blood supply to the placenta is minimal; oxygen tension in the placenta remains low (approximately 2% O_2_, a hypoxic state), and nutrients (e.g., 1 mM glucose) are scarce, much lower than levels in maternal circulation [44,45]. This low-oxygen, low-nutrient milieu persists until maternal blood flow is fully established by the end of the first trimester (around week 10), at which point placental oxygen rises to approximately 8% [45]. Despite these harsh conditions, EVTs successfully invade the decidua and superficial myometrium, remodeling uterine arteries to perfuse the placenta. The fact that EVTs penetrate up to one-third of the myometrium under hypoxic and nutrient-limited conditions, yet subsequently halt further invasion, highlights the presence of precisely regulated mechanisms controlling trophoblast invasion.

Importantly, recent spatial multi-omics define a sequence from villous cytotrophoblast-cell column cytotrophoblast (VCT-CCC) to EVT-1 and EVT-2, then divergence to endovascular EVT (eEVT) or interstitial EVT (iEVT), with terminal maturation as giant cells (GCs) [46]. Each step contributes a distinct function to placental morphogenesis. Progenitor states (VCT-CCC/EVT-1) sustain column growth and set downstream fate, as indicated by proliferative and progenitor programs (MKI67, NOTCH1). With transition to EVT-2, cells acquire firm adhesion and endothelial-like engagement with decidual matrix and vessel wall (ITGA1, CDH5). The lineage then bifurcates. iEVTs promote decidual and superficial myometrial infiltration via RAC1-driven motility and HLA-G-mediated immune crosstalk, while SERPINE1 tunes pericellular proteolysis. In parallel, eEVTs execute spiral-artery conversion through luminal colonization and controlled matrix remodeling, consistent with NCAM1 and MMP12 expression and reduced TGF-β signaling. Terminal GCs consolidate invasion arrest and tissue integration, aligned with Ephrin B1 (EFNB1) elevation and colony-stimulating factor 1 receptor (CSF1R) reduction. Across these stages, the signatures are not merely labels; they encode the minimal machinery by which trophoblasts anchor the placenta and establish an immune-compatible, perfused interface between mother and fetus.

**Figure 2:**
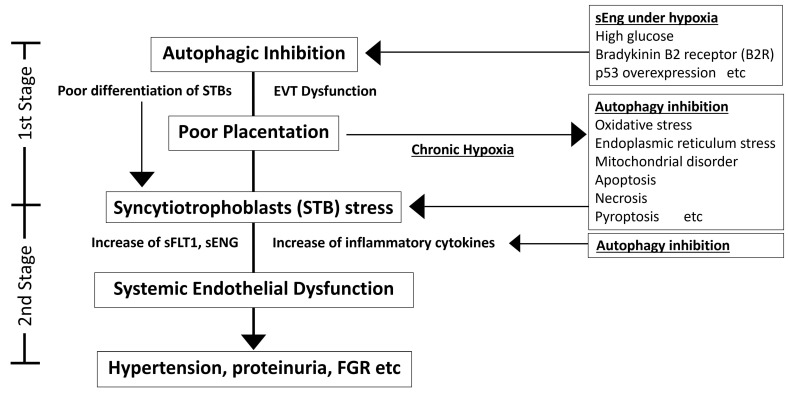
Revised Two-Stage Model of Preeclampsia Based on Autophagic Dysfunction. This figure presents a “Revised Two-Stage Model of Preeclampsia Based on Autophagic Dysfunction”, which augments the classical two-stage disorder theory by explicitly incorporating autophagy suppression as a mechanistic driver, with the aim of guiding future research into disease pathogenesis. Proposed model of how placental autophagy failure drives the two-stage pathway. Soluble endoglin (sEng) and p53 overexpression suppress autophagy in trophoblasts. Autophagy inhibition compromises extravillous trophoblast function and disrupts syncytiotrophoblast differentiation, leading to poor placentation and chronic placental hypoxia. Hypoxia amplifies oxidative and ER stress, mitochondrial dysfunction, and regulated cell-death programs, and it increases the release of anti-angiogenic factors (for example, sFlt1 and sEng) and inflammatory cytokines into the maternal circulation. These signals propagate systemic endothelial dysfunction that presents clinically as hypertension, proteinuria and FGR. A feed-forward loop is indicated: poor placentation sustains hypoxia, which further suppresses autophagy and intensifies syncytiotrophoblast stress. EVT, extravillous trophoblast; STB, syncytiotrophoblast; sFlt1, soluble fms-like tyrosine kinase-1; sEng, soluble endoglin; FGR, fetal growth restriction. The arrows in the figure indicate direction.

Failure of this regulated invasion, resulting in shallow EVT penetration, has dire consequences. Inadequate remodeling of maternal arteries leads to poor maternal blood flow to the placenta. The developing placenta, being underperfused, remains hypoxic and growth-restricted. This, in turn, triggers the release of stress signals, including inflammatory cytokines and anti-angiogenic factors, into the maternal circulation, which is outlined by the classic “Two-Stage model of preeclampsia” [47,48,49]. These circulating factors cause systemic maternal endothelial cell damage, manifesting as hypertension and end-organ dysfunction. Thus, shallow trophoblast invasion is a root cause of placental malformation and sets the stage for preeclampsia. In severe preeclampsia, the placenta is often small and underdeveloped, consistent with this scenario. To better understand why EVTs can invade so effectively under extreme conditions and why failure of invasion leads to this disease, we revisited the widely accepted “Two-Stage model”, hypothesis of pathophysiology leading to preeclampsia, and propose an extended framework: a “Revised Two-Stage model of preeclampsia based on Autophagic Dysfunction” (Figure 2). In this review, autophagy is posited as a key mechanism that enables EVTs to invade under hypoxic, nutrient-deprived conditions. Emerging evidence further suggests that early-gestational elevations of sEng can suppress autophagy in trophoblasts, weakening this adaptive program [5,50]. If autophagy in trophoblasts fails, EVTs cannot adequately invade or properly replace the endothelium of maternal spiral arteries, resulting in placental malformation and chronic placental hypoxia [5]. In the 2nd Stage, the hypoplastic and hypoxic placenta produces excessive stress factors, such as inflammatory molecules and anti-angiogenic proteins (like sFlt1), which enter maternal circulation and damage the maternal endothelium systemically [3]. This endothelial dysfunction culminates in the clinical syndrome of preeclampsia. In summary, normal placentation depends on deep EVT invasion into the decidua and myometrium; autophagy likely enables this under hostile conditions. The next section evaluates autophagy’s role in early placental physiology and its necessity for trophoblast invasion.

## 4. Autophagy in EVTs as Driving Forces in the Early Placental Milieu

Autophagy is a cellular self-digestion pathway activated by stressors such as nutrient starvation and hypoxia, precisely the conditions that occur during early placentation. Through autophagy, cells sequester cytoplasmic components (proteins or organelles) into double-membrane vesicles called autophagosomes, which then fuse with lysosomes to degrade the contents. This process recycles cellular building blocks and generates energy, helping to maintain metabolic balance under stress [51]. In early pregnancy, we hypothesized that autophagy serves as a vital survival mechanism for trophoblasts, enabling EVTs to withstand low oxygen and nutrient conditions during their invasive journey. Evidence from human placental tissue supports this idea. Autophagosomes, which were confirmed by Microtubule Associated Protein 1 Light Chain 3 beta (MAP1LC3B, hereafter LC3B) dot formation, have been observed in EVTs of first-trimester placentas (~8 weeks of gestation), indicating that autophagy is indeed active at the maternal–fetal interface during the period of deep trophoblast invasion [5]. Immunohistochemical staining for LC3B demonstrated the presence of distinct LC3B-positive puncta in EVTs deeply invading the maternal decidua. In contrast, such punctate signals were absent in EVTs located in the superficial decidual layer and in the overlying STBs. This suggests robust autophagosome formation in EVTs in situ, consistent with active autophagy in EVTs in vivo.

To directly test the role of autophagy in trophoblast function, researchers have employed trophoblast cell models with impaired autophagy. One key autophagy protein is Autophagy-related 4B cysteine peptidase (Atg4B), which is required for processing LC3 and autophagosome formation. Using a catalytically inactive ATG4B mutant (ATG4B^C74A^), which inhibits LC3B lipidation and hampers autophagosome closure, to create autophagy-deficient EVTs [52], ATG4B^C74A^ EVTs, despite similar proliferation, showed reduced matrix invasion exclusively under hypoxia, with normoxic invasion preserved [5] (Figure 2, Stage 1). Additionally, under chemically induced hypoxic conditions using cobalt chloride (CoCl_2_), which mimics severe low-oxygen stress, control EVTs maintained their invasive capacity, whereas ATG4B^C74A^ EVTs exhibited a markedly reduced ability to invade through a matrix, despite having normal proliferation rates [6]. Notably, the ATG4B^C74A^ EVTs failed to sustain intracellular ATP levels under CoCl_2_ treatment, whereas control EVTs preserved their energy homeostasis. This indicates that autophagy provides an emergency energy source for EVTs, likely by catabolizing intracellular substrates to fuel mitochondrial ATP production when external nutrients and oxygen are limited. By supplying energy in this manner, autophagy enables EVTs to power the energetically demanding process of migration and invasion. Beyond this role, evidence from glioblastoma has shown that inhibition of Serpin Family E member 1 (SERPINE1, also known as PAI-1), which is expressed in iEVTs as mentioned above, reduces cell survival and invasive capacity. SERPINE1 might be secreted through secretory autophagy, and autophagy appears to facilitate the release of active SERPINE1 into the extracellular space to promote invasion. In contrast, inhibition of autophagy leads to intracellular accumulation of inactive SERPINE1, thereby suppressing invasion [53]. Thus, suppression of autophagy under conditions such as chronic hypoxia could contribute to impaired EVT invasion via reduced SERPINE1 secretion. In this way, autophagy would be critically involved not only in providing metabolic energy but also in regulating molecular pathways that support the highly energy-demanding processes of EVT invasion. Consistent with this, EFNB1 expression increases in GCs once invasion ceases. EFNB1, a membrane-bound ligand for the Eph receptor B2 (EPHB2), mediates bidirectional signaling that regulates cell–cell interactions and boundary formation. Although EFNB1 has been reported to activate an autophagy-inducing pathway via ubiquitination of EPHB2 and LC3B recruitment [54], it remains unclear whether this mechanism operates in GCs. The interaction between trophoblastic EFNB1 and endothelial EPHB4 produces a repulsive signal that may redirect cellular energy from invasion to fusion [55], contributing to the cessation of trophoblast infiltration. The cytokines IL-34 and CSF-1, which are present at the maternal–fetal interface, also support the immunoregulatory functions of decidual macrophages and help maintain immune tolerance, yet low CSF1R expression in GCs may prevent them from activating autophagy, providing another potential explanation for the termination of EVT invasion [56].

Overall, autophagy serves as a critical adaptive mechanism that enables trophoblasts to endure and function within the hypoxic and nutrient-limited environment of early pregnancy. By sustaining cellular energy and quality control, it supports EVT survival, motility, and differentiation necessary for uterine invasion. When this process is impaired, invasion becomes shallow, leading to placental maldevelopment and subsequent maternal complications. Thus, autophagy not only safeguards trophoblast viability under metabolic stress but also ensures proper placentation, highlighting its potential as a therapeutic target for preventing preeclampsia.

## 5. The Role of Autophagy in Driving Syncytiotrophoblast Formation

Syncytialization of villous cytotrophoblasts is a critical differentiation process in which mononuclear cytotrophoblasts fuse and become multinucleated, undergo cell cycle arrest, and form the outermost layer of the villi. This structure mediates maternal–fetal exchange of nutrients and oxygen, enhances hormone secretion, and provides a barrier against infection [57]. Studies using BeWo cells have shown that syncytialization is associated with an increase in LC3B-II, suggesting that this process activates autophagy [58]. Proper expression of Endogenous Retrovirus group W member 1, envelope (ERVW-1, also known as Syncytin-1), Endogenous Retrovirus group FRD member 1, envelope (ERVFRD-1, also known as Syncytin-2), and suppressyn, along with cell cycle arrest, is essential for functional differentiation during syncytialization [57,59]. During syncytialization, autophagic activity was highest at the early stages of fusion; although LC3B-II levels remained elevated in fused BeWo cells, overall autophagic activity declined, as evidenced by the accumulation of Sequestosome 1 (p62/SQSTM1) during syncytialization [60]. Furthermore, syncytialization was significantly inhibited by autophagy inhibitors, with functional consequences including reduced human chorionic gonadotropin (hCG) secretion and impaired cell cycle arrest. Similar results were observed not only in BeWo cells but also in primary human trophoblasts and placental tissues. We also reported reduced Transcription Factor EB (TFEB) expression, a central regulator of autophagy [61], as an autophagy regulator in this context (Figure 2, Stage 1). Subsequent studies confirmed that TFEB expression is indispensable for syncytialization. ChIP-seq and functional analyses demonstrated that TFEB binds to the promoters of ERVFRD-1 and ERVW-1, enhancing their expression and promoting trophoblast fusion in BeWo cells, human trophoblast stem cells, and organoids. In contrast, TFEB deficiency markedly reduced syncytin expression and syncytialization [62,63]. Moreover, the Mammalian target of rapamycin complex 1 (mTORC1) inhibitor Torin-1 induced nuclear translocation of TFEB, increased syncytin expression and STB differentiation markers, and promoted syncytialization, whereas rapamycin, which does not induce nuclear translocation, showed no such effect, underscoring the importance of the mTORC1-TFEB nuclear translocation axis [62]. TFEB also regulates steroidogenic genes such as cytochrome P450 family 19 subfamily A member 1 (CYP19A1) (aromatase), thereby promoting estradiol production and enhancing hormone secretion in STBs [64]. Interestingly, despite TFEB deficiency, lysosome number, acidity, and proteolytic activity remained intact, suggesting that syncytialization failure is attributable not to impaired autophagy per se but rather to dysregulation of a distinct transcriptional program [63]. Clinically, both mRNA and protein levels of ERVW-1 are markedly reduced in villous trophoblasts of preeclamptic placentas [65]. In addition, hypoxia suppresses syncytin expression and trophoblast fusion in BeWo cells [66]. Importantly, reduced TFEB expression and nuclear TFEB localization have been observed in STBs of preeclamptic placentas, while chronic hypoxia decreases TFEB expression in primary human trophoblasts [16]. Collectively, reduced TFEB contributes to impaired syncytialization and poor placentation in preeclampsia.

## 6. Autophagy Deficiency Driving Impaired Placentation and Preeclampsia

Placental hypoxia and oxidative stress are central features in the pathogenesis of preeclampsia and related HDP [3]. In pregnancies that develop preeclampsia, the placenta often experiences chronic hypoxia, which is closely linked to increased ER stress, oxidative stress, inflammation, and trophoblast cell injury (Figure 2, Stage 1 to 2) [67]. This persistent hypoxic environment disrupts normal placental development and function, laying the groundwork for maternal hypertension and FGR. Autophagy, as a stress response mechanism, would normally be activated to mitigate hypoxic and oxidative damage by clearing damaged cellular components [68]. However, studies indicate that autophagic activity is impaired in preeclamptic placentas, exacerbating cellular stress. In other words, placental autophagy dysfunction not only results from chronic hypoxia but also contributes to placental malformation and the onset of preeclampsia. From the perspective of miscarriage, excessive oxidative stress during early pregnancy may inhibit villous development and angiogenesis, potentially leading to early pregnancy loss [69,70]. Excessive oxidative stress before or during early placental formation may cause miscarriage, while chronic oxidative stress after placental formation may lead to preeclampsia. However, the precise mechanisms underlying the differences in the relationship between autophagy and oxidative stress in the development of miscarriage versus preeclampsia remain unclear.

Biochemical evidence of autophagy impairment has been observed in placental tissues from women with preeclampsia. As indicative markers, p62, an autophagy substrate that binds to autophagosome membranes and is normally degraded, is often found to accumulate [16]. The accumulation of p62 suggests a blockade in autophagic flux: autophagosomes either are not forming adequately or are not being cleared via lysosomal degradation. This breakdown in the autophagy pathway means that cellular debris, damaged mitochondria, and protein aggregates are not efficiently removed. The result is heightened oxidative stress and inflammation within the placenta, as dysfunctional organelles (e.g., mitochondria producing reactive oxygen species (ROS)) and undegraded proteins trigger stress signaling. In essence, the preeclamptic placenta is stuck in a state of “autophagic dysregulation,” unable to restore homeostasis, which likely accelerates tissue damage and maldevelopment.

Causal evidence for autophagy’s role in placental health comes from animal models. A landmark study employed a conditional knockout approach to specifically disable autophagy in mouse trophoblasts by deleting the essential autophagy gene Atg7 in the placenta, utilizing two distinct trophoblast-specific Atg7 knockout lineages to validate the findings [7,71]. Molecular characterization of these two placenta-specific Atg7 knockout models revealed distinct phenotypes. In the trophoblast-specific Atg7 knockout (t-sKO), loss of Atg7 throughout the trophoblast population resulted in reduced LC3B-II protein levels and accumulation of p62, indicating impaired autophagic flux [71]. In contrast, the labyrinth-specific Atg7 knockout (l-sKO) showed no change in LC3B-II or Beclin1 expression (p62 was not reported) [71]. Notably, both models exhibited a common decrease in TFEB expression, suggesting a shared defect in lysosomal biogenesis and autophagy regulation [16,71]. Regarding placental and fetal growth, t-sKO placentas were significantly smaller, whereas fetal weights remained unchanged. In contrast, in the l-sKO, placental weights were comparable to controls, but fetal weights were significantly reduced. Morphologically, the t-sKO placentas showed a marked reduction in the spongiotrophoblast layer with no significant change in the labyrinth; whereas the l-sKO placentas demonstrated enlargement of the labyrinth layer without alteration in the spongiotrophoblast region. Although a comprehensive mechanistic analysis remains challenging, these findings imply that impaired autophagy in t-sKO placentas primarily affects the development of the spongiotrophoblast layer, leading to placental malformation and compensatory maternal hypertension that manifests as preeclampsia-like symptoms (Figure 2, Stage 1). In contrast, autophagy deficiency in l-sKO placentas likely contributes directly to FGR. Furthermore, as part of the biological stress response associated with preeclampsia, the l-sKO placentas exhibited a significant decrease in the antioxidant enzyme Superoxide dismutase 2 (SOD2) and multiple subunits of mitochondrial complexes, suggesting potential mitochondrial dysfunction. Autophagy functions not only as an intracellular degradation system but also as a regulator of nuclear transcription programs through interactions with key transcription factors and signaling pathways. We previously reported that autophagy inhibition reduces the expression of the transcription factor Nuclear factor erythroid 2-related factor 2 (Nrf2) and the antioxidant enzyme Heme Oxygenase-1 [72]. Recent reports indicate that reduced L-type amino acid transporter 1 (LAT1) is associated with decreased Nrf2 and an imbalance in angiogenic factors, reflected by an elevated sFlt-1/PlGF ratio, suggesting the LAT1-Nrf2 axis may be involved in the pathogenesis of preeclampsia [73]. Collectively, these findings suggest that autophagy inhibition could contribute to the pathogenesis of preeclampsia through dysregulation of Nrf2-mediated oxidative stress response, which secondarily leads to angiogenic imbalance. Collectively, the human and animal data underscore that proper autophagic flux in the placenta is required for normal trophoblast invasion and placental development, and when this process fails, it can initiate the cascade leading to preeclampsia. Taken together, these findings provide compelling evidence that defective placental autophagy plays a pivotal role in the initial stage of the “Revised Two-Stage model of preeclampsia based on Autophagic Dysfunction” in the pathogenesis.

At a molecular level, one major regulator of the trophoblast stress response is the transcription factor Hypoxia-Inducible Factor-1α (HIF-1α). Under the low-oxygen conditions of early pregnancy, HIF-1α is stabilized in trophoblast cells, promoting the expression of genes that facilitate adaptation to hypoxia. Notably, HIF-1α can induce autophagy as part of the cellular hypoxia response. In normal early gestation, HIF-1α activation in EVTs upregulates autophagy, which in turn supports trophoblast migration, invasion, and the vascular remodeling of spiral arteries [74]. This adaptive mechanism ensures that even in a hypoxic milieu, trophoblasts have sufficient energy and catabolic capacity to carry out placentation. However, in preeclampsia, the HIF-1α pathway appears dysregulated [75,76]. Placentas from preeclamptic pregnancies often show an inappropriate stabilization or timing of HIF-1α expression, which may be either excessive or prolonged, leading to maladaptive effects, or insufficient during the early window when it is required [77]. The disruption of normal HIF-1α dynamics could contribute to compromised autophagy in trophoblasts. Essentially, if HIF-1α signaling is disturbed, autophagy may not be properly induced at the critical time, exacerbating placental susceptibility to hypoxia [6,74,78]. This creates a vicious cycle: impaired autophagy leads to worsening placental hypoxia, which in turn drives further HIF-1α dysregulation and ultimately results in even greater suppression of autophagy. This self-perpetuating loop may serve as a critical link between the first stage of poor placentation and the second stage of systemic endothelial dysfunction in our hypothesis.

Another crucial piece in the puzzle of autophagy dysfunction is the protein Rubicon (Run domain Beclin-1-interacting cysteine-rich protein), a key negative regulator of autophagy. Rubicon associates with the autophagy machinery and inhibits the final step of the process, namely the fusion of autophagosomes with lysosomes, thereby blocking the degradation of autophagic cargo [79]. Under normal conditions, Rubicon levels are tightly regulated. Notably, studies from Yoshimori’s group have demonstrated that Rubicon accumulates with age in organs such as the liver and kidney, leading to suppression of autophagy and age-related functional decline [80]. More recently, increased Rubicon expression has been reported in postmenopausal ovaries, where it appears to contribute to the vulnerability of granulosa cells to oxidative stress [81]. Although similar accumulation has not yet been reported in the placenta, these findings raise the possibility that aberrant Rubicon upregulation could likewise contribute to autophagy failure in preeclampsia. In trophoblast cells already under hypoxic stress, excess Rubicon can further impede autophagic flux, leading to an accumulation of dysfunctional organelles and proteins. This buildup of cellular “garbage” can trigger trophoblast apoptosis and inflammatory signaling, compounding placental dysfunction. Alternatively, endothelial cells rely on basal autophagy to remove damaged mitochondria and regulate redox balance; when Rubicon inhibits this process, it results in mitochondrial accumulation and heightened oxidative stress in the endothelium [82]. Endothelial Atg5 deficiency is associated with impaired vasodilatory capacity and enhanced pressor responsiveness, reflecting global endothelial dysfunction. Such systemic and renal endothelial injury plausibly contributes to the cardinal features of preeclampsia, namely maternal hypertension and proteinuria [82]. Rubicon thus appears to create a vicious cycle wherein autophagy inhibition in both placenta and maternal endothelium reinforces oxidative and inflammatory damage, worsening the disease on both fronts. Given its central role, Rubicon or related inhibitory factors have emerged as a promising therapeutic target for preeclampsia. In theory, inhibiting Rubicon (or its interaction with the autophagy machinery) could reopen the autophagic pathway in trophoblasts and endothelial cells, allowing cells to clear harmful debris.

Under normal conditions, mitophagy selectively removes damaged mitochondria, thereby preventing the accumulation of intracellular ROS and alleviating oxidative stress. However, in the context of preeclampsia, whether activation of mitophagy is protective or detrimental remains controversial. One perspective suggests that impaired mitophagy contributes to the progression of disease. In placentas from preeclamptic patients, reduced expression of the mitophagy receptor B-cell/CLL lymphoma 2 (BCL2) interacting protein 3 (BNIP3) has been reported, leading to autophagy dysfunction and accumulation of damaged mitochondria, which in turn exacerbate pathology [21]. This view proposes that insufficient clearance of damaged mitochondria results in ROS accumulation, thereby amplifying inflammation and cell death. Consistently, reduced activity of the PTEN-induced kinase 1 (PINK1)/parkin RBR E3 ubiquitin protein ligase (PRKN) pathway has also been documented in preeclamptic placentas, accompanied by swollen, abnormal mitochondria and enhanced inflammatory cell death (pyroptosis). In trophoblasts subjected to hypoxia-reoxygenation stress, decreased mitophagy further aggravates pyroptosis, whereas PINK1 overexpression significantly reduces ROS production and pyroptosis, indicating a protective role for mitophagy against oxidative stress [18]. Additional evidence shows that macroautophagy-mediated degradation of inflammasomes can suppress pyroptosis under hypoxia-reoxygenation stress, highlighting the anti-inflammatory functions of autophagy (Figure 2, Stage 2) [67]. Ausman and colleagues further reported mitochondrial fragmentation and enhanced mitophagy associated with ceramide (CER) accumulation in PE placentas. CER promotes phosphorylation of the mitochondrial fission factor Dynamin 1 like (DNM1L) via BOK (Bcl-2-related ovarian killer), while reducing the fusion factor Optic Atrophy 1 (OPA1) [83]. PE placentas also show accumulation of PINK1 and PRKN, suggesting that CER/BOK/DNM1L-induced mitochondrial fragmentation leads to oxidative stress, but fragments are subsequently cleared through PINK1/PRKN-dependent mitophagy. The same group also demonstrated that exosomes released from preeclampsia-derived stromal cells under hypoxia can promote mitochondrial fission and induce cell death in surrounding trophoblasts [84]. Altogether, these findings support the concept that insufficient mitophagy, by allowing dysfunctional mitochondria to accumulate, contributes to ROS production, trophoblast toxicity, and disease progression, which is consistent with the canonical view of mitophagy as a protective process.

In contrast, other studies argue that excessive or dysregulated mitophagy worsens pathology. Enhanced mitochondrial fission in preeclamptic placentas is associated with increased mitophagy, which has been linked to excessive trophoblast death. Mechanistically, in preeclamptic model mice and EVTs under hypoxia, non-ubiquitinated FUN14 domain containing 1 (FUNDC1), which localizes to the outer mitochondrial membrane and mediates hypoxia-induced mitophagy via LC3B interaction [85], has been shown to promote mitophagy, resulting in oxidative stress aggravation and cell injury [86]. Inhibition of mitophagy in this setting alleviated cellular damage, suggesting that mitophagy hyperactivation may be deleterious. Other reports have also described increased BNIP3 and BNIP3L, decreased FUNDC1, and elevated p62 in preeclamptic placentas, changes that can lower mitochondrial membrane potential and activate autophagy/apoptosis pathways, thereby driving trophoblast cell death and disease progression [87]. Electron microscopy consistently reveals swollen or fragmented abnormal mitochondria in preeclamptic placentas, indicative of mitochondria with depolarized membranes and impaired function. Whether these mitochondrial changes represent a continuous process or distinct pathogenic events remains unclear, contributing to the complexity of interpretation. Normally, cells rely on mitophagy to remove such dysfunctional mitochondria and suppress ROS generation. While this process is beneficial when mitophagy functions normally, the placental environment in preeclampsia is characterized by chronic hypoxia, which leads to an autophagy-exhausting condition, along with disturbed mitochondrial quality control (e.g., excessive mitochondrial fragmentation and ROS accumulation). As a result, damaged mitochondria accumulate, and mitophagy itself becomes dysregulated, leading to mitochondrial imbalance and heightened oxidative stress. Experimental findings support this duality: under hypoxia, inhibition of mitophagy in trophoblasts can actually reduce ROS levels and cellular injury, suggesting that excessive mitophagy may be harmful. Thus, mitophagy in preeclampsia presents a paradoxical and dual nature, being both protective and potentially detrimental depending on the context. Understanding its role in disease will require accurate methods to assess mitophagy activity. Without such precise evaluation, it cannot be concluded that simple inhibition or activation of mitophagy would improve placental function.

In summary, placental autophagy dysfunction, arising from genetic deficits (e.g., Atg7 loss), signaling abnormalities (such as HIF-1α dysregulation), mitochondria-related oxidative stress (e.g., impaired mitophagy), or the accumulation of autophagy inhibitors such as Rubicon, plays an important role in the pathophysiology of preeclampsia and other HDP. These insights connect the dots between placental hypoxia, failed cellular adaptation (autophagy), and the maternal syndrome of preeclampsia. By uncovering these molecular links, we open the door to novel diagnostic and therapeutic strategies aimed at the root causes of HDP, rather than just managing its symptoms.

## 7. Therapeutic and Diagnostic Prospects

Recognition of autophagy’s role in placental development and preeclamptic pathogenesis suggests new opportunities for intervention. Rather than treating preeclampsia only after clinical onset, we might target the underlying autophagic dysfunction to prevent or mitigate the disease. Several therapeutic avenues are under exploration.

Pharmacological Enhancement of Autophagy: One approach is to boost autophagy in the placenta using drugs. mTOR is a master negative regulator of autophagy; thus, mTOR inhibitors like rapamycin can induce autophagy in many tissues. Similarly, activation of AMP-activated protein kinase (AMPK), a cellular energy sensor, promotes autophagy; the antidiabetic drug metformin is an AMPK activator with a good safety profile. Both rapamycin and metformin have shown the ability to increase autophagic activity in various models [88,89] and could potentially be repurposed to improve placental autophagy in pregnancies at risk for preeclampsia. By restoring autophagic flux, these therapies may reduce placental hypoxic stress and improve trophoblast function, thereby preventing or ameliorating the development of maternal hypertension and FGR. It is important to note, however, that any drug used in pregnancy must be evaluated carefully for fetal safety. Rapamycin, for instance, is a potent immunosuppressant and anti-proliferative agent, which might raise concerns during pregnancy; lower doses or localized delivery to the placenta could be considered. Pregnant dams received rapamycin injections every 12 h from 15.5 days post coitum until delivery, resulting in an approximately 16% reduction in neonatal body weight [90]. Metformin, on the other hand, is already used in gestational diabetes and has shown some benefits in preeclampsia prevention in clinical studies, possibly via improved placental function. In rat models of lipopolysaccharide-induced or NG-nitro-L-arginine methyl ester (L-NAME)-induced preeclampsia, metformin significantly ameliorated FGR and maternal hypertension, without affecting fetal weight in control animals [91,92].

Targeting HIF-1α Pathway: As discussed, HIF-1α is a key driver of trophoblast autophagy and invasion under physiological hypoxia. In preeclampsia, insufficient HIF-1α activity in early pregnancy could contribute to shallow invasion. Conversely, enhancing HIF-1α signaling in the trophoblast (within a normal range) might promote autophagy and healthy invasion. Stabilizing HIF-1α pharmacologically or via gene therapy is an intriguing strategy. For example, small molecules that inhibit prolyl hydroxylases, which mark HIF-1α for degradation, could prolong HIF-1α activity. Care would be needed, since excessive HIF-1α can also be pathological, but a transient boost in early gestation might improve placentation. However, a key challenge lies in identifying patients who already exhibit inadequate EVT invasion at the early stage of pregnancy. Moreover, excessive invasion beyond the so-called ‘one-third myometrium enigma’ could lead to pathological conditions such as placenta percreta, underscoring the dual-edged nature of attempts to restore invasive capacity.

Autophagy-Related Biomarkers: On the diagnostic front, autophagy presents new possibilities for early detection and risk stratification of preeclampsia. If placental autophagic dysfunction precedes the clinical symptoms of preeclampsia, it could be detectable through biomarkers in maternal circulation. Circulating levels of autophagy-related proteins, such as LC3B-II, p62, and Beclin-1, in the peripheral blood mononuclear cells of pregnant women might be available for detecting preeclampsia [93]. Elevated levels of p62 or altered LC3B ratios in maternal blood might reflect inadequate autophagy in the placenta (since damaged cells might release contents or extracellular vesicles containing such proteins). Small placental-derived vesicles (exosomes) in the maternal bloodstream could also carry signatures of autophagic activity. Recent studies suggest that the identification of autophagy-related microRNAs may enable the prediction of preeclampsia [94,95]. The hope is that a panel of autophagy biomarkers could be measured in early pregnancy (for example, at the end of the first trimester) to predict which pregnancies are at high risk for developing preeclampsia. However, several important challenges remain before such approaches can be implemented in clinical practice. Furthermore, although longitudinal evaluation of autophagy activity throughout pregnancy, rather than a single-point measurement, may improve the prediction of preeclampsia onset, a major limitation currently exists in this field. At present, there is no clinically established and reproducible method for objectively evaluating autophagy activity in clinical samples. This limitation makes it difficult to determine the optimal timing for assessment in clinical practice. Therefore, future research should focus not only on clarifying the temporal dynamics of autophagy during pregnancy but also on developing simpler, clinically applicable methods for evaluating autophagy activity. If validated in large cohorts, such biomarkers would enable closer monitoring or prophylactic interventions (like low-dose aspirin, antioxidants, or future autophagy-targeted drugs) in high-risk individuals. In the future, in addition to the sFlt1/PlGF ratio, it is expected that candidate biomarkers such as those mentioned above will be identified, normative ranges will be established in uncomplicated pregnancies, and their predictive accuracy will be thoroughly evaluated.

## 8. Trehalose: A Naturally Derived Autophagy Activator

A particularly promising therapeutic approach involves naturally derived compounds that activate autophagy, which are attractive due to their potentially lower toxicity in pregnancy. One such compound is trehalose, a naturally occurring disaccharide sugar. Trehalose has garnered interest for its ability to induce autophagy through activation of TFEB, a master regulator of lysosomal biogenesis and autophagy pathways. By activating TFEB, trehalose can stimulate the production of lysosomal enzymes and promote the clearance of cellular waste [96]. In models of neurodegenerative diseases like Alzheimer’s, trehalose has shown therapeutic benefits, improving the clearance of toxic protein aggregates and rescuing cell function [97,98]. There is some debate about the mechanism: it is unknown whether trehalose directly activates TFEB [99]. However, multiple studies support its pro-autophagy effects and neuroprotective outcome. These findings have spurred exploration of trehalose beyond neurology, into conditions like preeclampsia, where enhanced autophagy might be beneficial. In the context of preeclampsia, trehalose offers several advantages for the usage to pregnant women. First, it is a non-toxic, naturally occurring sugar that has been used as a food additive and even tested in humans for other indications, suggesting a favorable safety profile. During pregnancy, safety is paramount; therapies that do not harm the fetus are required. In this context, an IL-10 knockout (IL-10^−^/^−^) preeclamptic mouse model, which is characterized by inflammation-driven preeclampsia-like symptoms, was used to test oral supplementation with trehalose as a therapeutic intervention [96]. In this model, administration of human preeclampsia serum to pregnant IL-10^−^/^−^ mice induces a preeclamptic phenotype. Remarkably, trehalose treatment led to significant improvement: compared with untreated preeclamptic mice, systolic blood pressure was markedly reduced, and the proteinuria was also improved to near-normal levels. These suggest that trehalose mitigated endothelial dysfunction and kidney injury.

## 9. Preeclampsia’s Long Shadow: Implications for Neurodegenerative Disorders

An intriguing aspect of autophagy dysfunction in preeclampsia is its potential connection to diseases later in the mother’s life, particularly neurodegenerative diseases from the viewpoint of autophagy. It is well-documented from animal and cellular models that impairing autophagy in the central nervous system can lead to neurodegeneration. For example, mice with neural-specific knockout of autophagy genes (such as Atg5 or Atg7) exhibit progressive neurodegenerative changes, including accumulation of protein aggregates and neuronal loss [100,101]. Hara et al. famously showed that mice lacking Atg5 in neural cells developed ubiquitin-positive inclusion bodies in neurons and had motor deficits, essentially recapitulating aspects of neurodegenerative disease [100]. Furthermore, genetic studies in humans have linked autophagy-related genes to neurological disorders: mutations in PINK1 and PRKN are associated with familial Parkinson’s disease, implicating defective mitophagy in its pathogenesis, and polymorphisms in autophagy genes like ULK1 and ATG16L1 have been linked to Crohn’s disease and might also influence neuroinflammatory conditions [102,103]. These examples illustrate that autophagy dysfunction is a common pathological thread across diverse conditions ranging from inflammatory bowel disease to neurodegeneration.

Given this background, researchers have hypothesized that the occurrence of severe preeclampsia might predispose women to neurodegenerative diseases in later life. Women who experience preeclampsia are already known to have a higher risk of long-term cardiovascular disease and stroke [104,105]. Could there also be a link to dementia or other neurodegenerative disorders? A recent systematic review and meta-analysis by some investigators clarified this question and found a significant association: women with a history of preeclampsia or eclampsia had about a 1.4-fold increased risk of developing Alzheimer’s disease and an even greater 3.1-fold increased risk of vascular dementia later in life, compared to women with normotensive pregnancies [102,106]. These findings suggest that HDP/preeclampsia is an important risk factor for maternal cognitive decline in aging, especially for forms of dementia related to vascular pathology.

The mechanistic link between preeclampsia and neurodegeneration may lie in systemic endothelial dysfunction and inflammation, processes common to both conditions. Preeclampsia is characterized by widespread maternal endothelial injury (the linings of blood vessels throughout the body are damaged, leading to hypertension and organ ischemia). In fact, the clinical diagnosis of preeclampsia hinges on signs of endothelial leak (proteinuria) and high blood pressure. Similarly, many neurodegenerative diseases, particularly vascular dementia and even Alzheimer’s, involve chronic cerebrovascular dysfunction—damage to the brain’s blood vessels and microcirculation leads to impaired clearance of toxins, blood–brain barrier breakdown, and neural injury. It is plausible that women who have preeclampsia sustain a subclinical insult to their vascular endothelium (including cerebral endothelium) during the episode of preeclampsia, and that this injury does not entirely resolve after pregnancy. Over the years or decades, this could leave the brain more vulnerable to neurodegenerative changes. Autophagy might serve as a connecting mechanism: the same autophagy failure that contributes to placental and endothelial stress in preeclampsia could, in an aging individual, lead to less efficient clearance of protein aggregates or damaged mitochondria in the brain. Furthermore, there may be shared genetic alterations affecting vascular endothelial integrity that link preeclampsia and neurodegenerative disorders.

## 10. Conclusions

Preeclampsia remains a disease surrounded by competing theories, reflecting the multifactorial and enigmatic nature of its pathogenesis. This phrase underscores the numerous and competing hypotheses proposed to explain preeclampsia, which are explained for immunological maladaptation, placental ischemia, and systemic endothelial dysfunction. Likewise, preeclampsia can be regarded as “a field of complex classifications,” given its widely varying clinical presentation and severity, which necessitate ongoing refinements in diagnostic criteria and subclassifications (early-onset vs. late-onset, with or without severe features, etc.). Within this complex mosaic, our review highlights placental autophagy dysfunction as a unifying mechanistic thread that links the abnormal placental development (shallow trophoblast invasion, malperfusion) with the maternal syndrome of hypertension and organ injury. Autophagy is not the sole explanation for preeclampsia; rather, it interacts with immune, genetic, and environmental factors, and understanding its role provides a cohesive framework that integrates many observations (e.g., hypoxia, oxidative stress, failed spiral artery remodeling, and even long-term maternal risks). From this perspective, an important direction for research and clinical practice is the development of methods to detect placental autophagy dysfunction in vivo and to determine when and to what extent autophagy derailment contributes to human preeclampsia. Early detection could be achieved through biomarkers, as discussed, or advanced imaging of placental function. If we can identify the onset of placental stress (e.g., rising p62 levels or altered metabolite profiles in maternal blood), it opens a window for timely intervention, which potentially rescues the pregnancy before severe preeclampsia develops. Ultimately, the goal is to translate these mechanistic insights into therapies that improve outcomes for pregnant women and their children. For example, a future standard of care might include an autophagy-enhancing supplement in prenatals for high-risk mothers, or an inhibitor of autophagy-inhibitory factors delivered via nanoparticles to the placenta for those showing early signs of trouble. Such interventions, while aspirational now, underscore a shift toward precision medicine in obstetrics, where we treat the placenta to treat the mother. Realizing this vision will require interdisciplinary collaboration, including obstetricians, placental biologists, pharmacologists, and epidemiologists working together to ensure that laboratory findings successfully translate to improved outcomes at the bedside. Nonetheless, the prospect of addressing the root causes of preeclampsia is an exciting paradigm shift. By focusing on the health of the placenta, we can aim for safer pregnancies and healthier mothers and babies.

In conclusion, placental autophagy sits at the crossroads of trophoblast biology, maternal–fetal medicine, and even long-term maternal health. By advancing our understanding of autophagy in the context of pregnancy, we not only move closer to unraveling the mystery of preeclampsia’s origins but also pave the way for innovative strategies to ensure healthier pregnancies and healthier lives for mothers beyond pregnancy.

## Figures and Tables

**Table 1:** Reports Suggesting Autophagy Activation in Preeclampsia.

Author (Year)	Model	Autophagy-Related Molecules	Findings
Oh et al. (2008) [13]	Human placenta	LC3-II	Increased LC3-II
Akaishi et al. (2014) [8]	Human placenta with hypertension	LC3-II P62	Increased LC3-II Decreased p62
Hutabarat et al. (2017) [10]	Human placenta	LC3-II Beclin-1	High expression in early-onset preeclampsia
Öcal et al. (2023) [12]	Human placenta	Beclin-1	Beclin-1 increases in decidua and villi
García-Puente et al. (2024) [9]	Human placenta	ULK1 ATG5 ATG9A LC3 LAMP1	Autophagy-related factors increase in late-onset preeclampsia
Ma et al. (2024) [11]	RUPP rat model	Autophagy pathway genes	Elevated autophagy-related gene expression in the preeclampsia placenta.
Zhao et al. (2025) [14]	Human placenta L-NAME mouse model	BNIP3 NLRP1	BNIP3-mediated mitophagy activated inflammasome

**Table 2:** Reports Suggesting Autophagy Inhibition in Preeclampsia.

Author (Year)	Model	Autophagy-Related Molecules	Findings
Aoki et al. (2018) [7]	Trophoblast-specific Atg7 KO mice	Atg7 p62 LC3	p62 accumulation in the placentas of preeclampsia model mice
Nakashima et al. (2020) [16]	Human placentas Trophoblast cells Atg7 KO mice	LC3-II LAMP1 TFEB	TFEB-mediated lysosomal dysfunction suppresses autophagy
Zhou et al. (2021) [21]	Human placenta Trophoblast cells	BNIP3 LC3-II Beclin-1 p62	BNIP3 decreased in preeclampsia placenta
Ribeiro et al. (2022) [17]	Human placenta	p62	p62 accumulation indicates impaired degradation
Cheng et al. (2022) [15]	Primary trophoblasts	Atg5-Atg12 LC3-II GABARAP	Autophagy-lysosome machinery impaired
Weel et al. (2023) [19]	Human placentas	LC3 Beclin-1 mTOR	mTOR-mediated autophagy suppression
Sun et al. (2023) [18]	Human placenta	PINK1	PINK1-mediated mitophagy suppressed
Zhou et al. (2024) [20]	Human placenta Literature synthesis	Beclin-1 LC3B p62	Suppression of autophagy is associated with preeclampsia

## Data Availability

No new data were created or analyzed in this study. Data sharing is not applicable to this article.

## Abbreviations

The following abbreviations are used in this manuscript:

AMPK	AMP-Activated Protein Kinase
ATG4B	Autophagy-related 4B cysteine peptidase
BCL2	B-cell/CLL lymphoma 2
BNIP3	BCL2-interacting protein 3
BOK	Bcl-2-related ovarian killer
CoCl_2_	Cobalt chloride
CSF1R	Colony-stimulating factor 1 receptor
CYP19A1	cytochrome P450 family 19 subfamily A member 1
DNM1L	Dynamin 1-like
EFNB1	Ephrin B1
EPHB2	EPH receptor B2
EPHB4	EPH receptor B4
ER	Endoplasmic Reticulum
ERVFRD-1	Endogenous Retrovirus group FRD member 1
ERVW-1	Endogenous Retrovirus group W member 1
EVT	Extravillous trophoblast
FGR	Fetal growth restriction
FUNDC1	FUN14 domain containing 1
GCs	giant cells
hCG	human chorionic gonadotropin
HDP	Hypertensive disorders of pregnancy
HIF-1α	Hypoxia-inducible factor-1 alpha
LAT1	L-type amino acid transporter 1
MAP1LC3B	Microtubule-associated protein 1 light chain 3 beta
mTORC1	Mammalian (mechanistic) target of rapamycin complex 1
Nrf2	Nuclear factor erythroid 2-related factor 2
SERPINE1	Serpin family E member 1
SOD2	Superoxide dismutase 2
OPA1	Optic Atrophy 1
PINK1	PTEN-induced kinase 1
PlGF	Placental growth factor
PRKN	Parkin RBR E3 ubiquitin protein ligase
p62/SQSTM1	Sequestosome 1
ROS	Reactive oxygen species
Rubicon	Run domain Becline-1-interacting cysteine-rich protein
sEng	Soluble endoglin
sFlt1	Soluble fms-like tyrosine kinase-1
STB	Syncytiotrophoblast
TFEB	Transcription factor EB
TGF-β	Transforming growth factor-β
ULK1	uni-51-like autophagy activating kinase 1
VCT-CCC	villous cytotrophoblast-cee column cytotrophoblast
